# Health-Related Quality of Life (HRQoL) Among Adults With Non-communicable Diseases in a Selected District of South India: A Cross-Sectional Study

**DOI:** 10.7759/cureus.91006

**Published:** 2025-08-26

**Authors:** Shankar Radhakrishnan, Megavannan Thamizhkovan, Raju Kannan, Sangeetha S

**Affiliations:** 1 Family Medicine, Vinayaka Mission's Kirupananda Variyar Medical College and Hospitals, Salem, IND; 2 Community Medicine, Vinayaka Mission's Kirupananda Variyar Medical College and Hospitals, Salem, IND

**Keywords:** community-based study, cross-sectional study, eq-5d-5l, health-related quality of life, noncommunicable diseases

## Abstract

Background

Non-communicable diseases (NCDs) contribute significantly to long-term morbidity and reduced well-being. Understanding health-related quality of life (HRQoL) among NCD patients is essential for context-specific healthcare planning. This study evaluates HRQoL among NCD patients in a semi-urban district in Tamil Nadu and examines its association with socio-demographic factors.

Methods

A cross-sectional study was conducted in the Family Adoption Area of a Medical College in South India between November 2024 and January 2025. A sample size of 364 was calculated, and NCD patients identified in that area who fulfilled the eligibility criteria were included in the study by systematic random sampling. The standardized study tool, EQ-5D-5L, was used to assess HRQoL. Collected data were entered into MS Excel 2019 (Microsoft® Corp., Redmond, WA, USA) and analyzed using IBM SPSS Statistics for Windows, Version 21 (Released 2012; IBM Corp., Armonk, NY, USA). Associations were determined using t-tests and multiple linear regression.

Results

A total of 364 participants were recruited, with a mean age of 52.2 years and an almost equal distribution between both sexes. The participants without any problems in the domains of HRQoL - which include mobility, self-care, usual activities, pain/discomfort, and anxiety/depression - were 60.7%, 61.6%, 57.7%, 37.4%, and 70.4%, respectively. The mean EQ index score of all the participants was 0.86 ± 0.19. Significant predictors of poor HRQoL included age ≥ 60 years, being single or widowed, having non-formal or primary education, and a disease duration >3 years (R² = 0.231).

Conclusion

Around one-third of the NCD patients had varying degrees of problems in the different domains of HRQoL, with pain/discomfort being the most affected. Targeted interventions, such as pain management, health literacy programs, and elderly psychosocial support, should be integrated into NCD care strategies.

## Introduction

Non-communicable diseases (NCDs) are the leading global health challenge, contributing significantly to morbidity and mortality profiles [1]. The World Health Organization states that, globally, more than 74% of all deaths are due to NCDs [2]. Its burden is relatively higher in low- and middle-income countries, whereas in India, the proportional mortality rate accounted for by NCDs is 63% [3]. According to the National Programme for Prevention and Control of Non-Communicable Diseases (operational guidelines 2023-2030), the common NCDs in India are diabetes (65 million), chronic obstructive pulmonary diseases (55 million), cardiovascular diseases (54.5 million), and cancer (3.2 million based on GLOBOCAN5) [4,5]. These data support the evidence that India is undergoing a significant epidemiological transition marked by a shift from communicable diseases to NCDs [6]. This transition is accompanied by an increase in life expectancy, with the average lifespan rising from 55 years to 67.3 years, reflecting advancements in healthcare [7]. The increase in life expectancy and the rise in NCDs have created a significant burden of Disability-Adjusted Life Years (DALYs) [8]. This growing burden has a profound adverse impact on health-related quality of life (HRQoL), as individuals live longer with chronic conditions that affect their physical, mental, and social well-being.

HRQoL is a multidimensional concept that encompasses an individual's subjective perception of the impact of health status, including the disease and treatment, on physical, psychological, and social aspects of life [9]. The burden of NCDs extends beyond clinical outcomes and biomedical measures to affect patients' emotional health, performance of daily essential activities, social participation, and economic stability. It is a useful indicator of overall health, and there are different tools, like EuroQoL 5D-5L and the 36-item Short-Form (SF-36) survey, to assess it [10]. Evaluating HRQoL provides a more comprehensive understanding of the disease's impact on health status. Despite the growing recognition of HRQoL as a critical indicator in assessing health status, there is limited research on its determinants in our study setting. Therefore, this study aims to estimate HRQoL among NCD patients and assess the factors influencing it in the Salem district of Tamil Nadu. The study location in Salem offers a rural demographic with limited research on community-based HRQoL, making it an important area for study. The findings from this study can help policymakers design effective strategies to optimize the well-being of NCD patients. 

Objectives

The objectives of the study were to estimate the HRQoL among the NCD patients of Salem district, Tamil Nadu, and assess its association with socio-demographic characteristics using EQ-5D-5L index scores of the study participants.

## Materials and methods

A community-based cross-sectional study was conducted between November 2024 and January 2025 among the NCD patients. The Family Adoption Program was implemented by Vinayaka Mission's Kirupananda Variyar Medical College and Hospitals, Salem, India, in our designated field practice area of Neikarapatti, Salem, from which around 750 houses were enlisted (approval no. VMKVMC&H/IEC/25/013). The data of the NCD patients from the health records were extracted and considered as the sampling frame for this study. For this study, the most common NCDs in the area were included. These are diabetes mellitus, hypertension, cardiovascular diseases (like ischemic heart disease and stroke), and chronic respiratory diseases (like chronic obstructive pulmonary disease and asthma). The inclusion criteria for participants were being 18 years or older, having a doctor-confirmed diagnosis of at least one of the above NCDs for at least six months, and living in the study area for a minimum of one year. The exclusion criteria for participants were those with cognitive impairments, having severe psychiatric conditions that would make an interview impossible, or being critically ill. NCD diagnoses were extracted from individual household health records maintained under the Family Adoption Program and confirmed via physician-validated documentation, such as treatment cards, hospital discharge summaries, and laboratory reports. Patients with ambiguous or self-reported-only diagnoses were excluded to ensure validity. Administrative approvals to assess the records and ethical approval to conduct the study were obtained before the start of the study.

Considering the proportion of 64.5% (Level 1/no problem in the domain of anxiety and depression of EQ-5D-5L) from the study by Parik and Patel among diabetics [11], with an absolute precision of 5%, the final sample size estimated was 364. A master list of all eligible NCD patients from the 750 enlisted houses served as the sampling frame. Participants were selected from this master list using a systematic random sampling technique. The sampling interval was calculated by dividing the total number of eligible patients by the required sample size. If a household contained more than one eligible individual, both were included in the sampling frame, ensuring each person had an equal chance of selection. The selected participants were visited in their homes and interviewed by trained medical interns using a semi-structured questionnaire (see Appendix). The questionnaire was translated into the local language (Tamil) and then back-translated into English to ensure accuracy. The study details were explained to the participants, and written informed consent was obtained before recording the data. If the selected participant was not available even after two visits, then the next enlisted eligible participant from the sampling frame was selected. A total of 380 individuals were approached, out of which 364 consented to participate, yielding a response rate of 95.8%. The final sample size of 364 was achieved, slightly exceeding the calculated minimum of 362.

The questionnaire comprises two sections, with the first part covering socio-demographic characteristics, including age, gender, education, occupation, marital status, family members, monthly income, morbidity status, duration of the NCD morbidity, and addiction behavior. The socio-economic status was classified using the Modified BG Prasad Scale [12], updated for the All India Consumer Price Index (AICPI) for January 2025.

The second section assessed HRQoL using the validated European Quality of Life 5-Dimension 5-Level (EQ-5D-5L) questionnaire. This instrument evaluates five domains: mobility, self-care, usual activities, pain/discomfort, and anxiety/depression. For each domain, participants describe their health state by choosing one of five severity levels: Level 1 (no problems), Level 2 (slight problems), Level 3 (moderate problems), Level 4 (severe problems), and Level 5 (extreme problems/unable to perform). The responses from the five domains are combined to create a five-digit number that represents the participant's unique health state (e.g., "11111" indicates no problems in any domain, while "21121" indicates slight problems in mobility and pain/discomfort and no problems in the other domains). To calculate a single summary score for HRQoL, this health state is converted into an EQ-Index value.

This study used the official EQ-5D-5L value set developed for India, which assigns a specific weight to each health state, mapping it onto a scale where 1 represents perfect health and 0 represents death [13]. There is no specific cut-off for "good" or "poor" HRQoL; rather, the index is a continuous variable where lower scores indicate poorer HRQoL. The last question was on the EQ Visual Analog Scale (VAS), scored between 0 and 100, where 100 indicates best and 0 indicates worst. The participant was asked to self-rate on the VAS by marking "X" to indicate his/her current health status. The Cronbach’s alpha for the EQ-5D-5L questionnaire in the Indian setting among diabetic patients was 0.759 [11].

The data were entered in MS Excel 2019 (Microsoft® Corp., Redmond, WA, USA) and analyzed using IBM SPSS Statistics for Windows, Version 21 (Released 2012; IBM Corp., Armonk, NY, USA). Continuous data were represented as mean with standard deviation (SD) or median (inter-quartile range), and categorical data as proportions. The factors associated with the HRQoL of the participants were assessed using the calculated mean EQ index by applying Student's t-test. Factors found significant in univariate analysis were subjected to multiple linear regression, and the fitness of the model was analyzed using the R-squared value. A p-value <0.05 was considered statistically significant.

The assumptions for the multiple linear regression model were verified prior to analysis. The normality of residuals was assessed using a Q-Q plot, which showed the points falling approximately along the diagonal line. Linearity and homoscedasticity were checked by examining a scatterplot of the standardized residuals versus the standardized predicted values, which showed no discernible pattern. Finally, multicollinearity was checked using the Variance Inflation Factor (VIF), and all predictor variables had VIF values below 5, indicating that this assumption was not violated.

## Results

Among the 364 study participants included, the majority were under 60 years of age - 257 (70.6%) - with a mean age of 52.2 (±13.2) years. There was almost equal representation from both genders. Around one-third of the participants, 138 (37.9%), had non-formal education. Occupational status indicated that 237 (65.1%) of the participants were not working, including homemakers, retirees, and students. Around two-thirds of the participants belonged to the lower (113, 31.0%) and lower-middle (122, 33.5%) socio-economic classes according to the modified BG Prasad Scale. Approximately 96 (26.4%) were smokers, and 76 (20.9%) consumed alcohol. Among the NCD participants recruited, diabetics were the highest group (172, 47.3%), followed by hypertensives (149, 40.9%). The socio-demographic characteristics of the study participants are shown in Table 1.

**Table 1:** Socio-Demographic Characteristics of the Study Participants (N = 364) *The Modified BG Prasad Scale classifies families based on per capita monthly income [12]. The income brackets for January 2025 were used for categorization.

Variable		n	%
Age (years)	< 60	257	70.6
	≥ 60	107	29.4
Gender	Male	184	50.5
	Female	180	49.5
Educational Status	Non-formal	138	37.9
	Primary	119	32.7
	Secondary	51	14
	High/Higher	32	8.8
	Graduates	24	6.6
Occupational Status	Working	127	34.9
	Not working	237	65.1
Marital Status	Married	320	87.9
	Single/Widow/ Widower/Divorced	44	12.1
Socio-Economic Status (Modified BG Prasad Scale)*	Upper	14	3.9
	Upper-middle	51	14
	Middle	64	17.6
	Lower-middle	122	33.5
	Lower	113	31
Living status	With family	348	95.6
	Non-family members	1	0.3
	Alone	15	4.1
Smoking	Yes	96	26.4
	No	268	73.6
Alcohol Status	Yes	76	20.9
	No	288	79.1
Morbidity	Diabetes	172	47.3
	Hypertension	149	40.9
	Cardiovascular Diseases	37	10.2
	Respiratory Diseases	6	1.6

In Table 2, HRQoL was analyzed using the EQ-5D-5L questionnaire, which includes five domains. The worst-affected domain was pain/discomfort, with only 136 (37.4%) reporting no problems (Level 1). The anxiety/depression domain was the least affected, with around two-thirds (256, 70.4%) reporting no problems (Level 1). The domain with the highest proportion of extreme problems (Level 5) was self-care, with 4 (1.1%) participants. The median EQ VAS was 75 (65-85). The mean EQ index score of all participants was 0.86 ± 0.19. The proportion of participants reporting good health - no problems in all five domains (“11111”) - was 113 (31%).

**Table 2:** Health-Related Quality of Life of the Study Participants (N = 364) *Levels indicate the severity of problems reported by participants: Level 1, no problems; Level 2, slight problems; Level 3, moderate problems; Level 4, severe problems; Level 5, extreme problems/unable to perform.

	Level 1, n (%)*	Level 2, n (%)	Level 3, n (%)	Level 4, n (%)	Level 5, n (%)
Mobility	221 (60.7)	115 (31.6)	25 (6.9)	2 (0.5)	1 (0.3)
Self-Care	224 (61.6)	109 (29.9)	23 (6.3)	4 (1.1)	4 (1.1)
Usual Activities	210 (57.7)	119 (32.7)	29 (7.9)	4 (1.1)	2 (0.5)
Pain/Discomfort	136 (37.4)	171 (47.0)	50 (13.7)	5 (1.4)	2 (0.5)
Anxiety/Depression	256 (70.4)	86 (23.6)	19 (5.2)	3 (0.8)	0 (0)

Table 3 represents the univariate analysis. The factors that include age ≥ 60 years; non-formal or primary level education (Class I to Class V); not working; being single, widow, widower, or divorced; belonging to lower-middle and lower socio-economic classes; and duration of NCD morbidity >3 years had lower mean EQ index values. These differences were found to be statistically significant (p-value < 0.05).

**Table 3:** Association Between Participants Characteristics and EQ Index Value (N = 364) Student's t-test was used to compare EQ index values between groups. The table shows mean values with corresponding t-values and p-values. The "Others*" category represents the combined group of participants with higher levels of education. Specifically, it includes everyone who reported their educational status as Secondary, High/Higher, or Graduates. This grouping was done to compare the HRQoL of participants with non-formal or primary education against those with secondary education or higher. The Modified BG Prasad Scale classifies families based on per capita monthly income [12]. The bold values highlight the p-values that were statistically significant (i.e., p < 0.05).

Variables		Mean	Standard Deviation	t-value	p-value
Age (years)	< 60	0.91	0.12	5.86	<0.001
	≥ 60	0.74	0.29		
Gender	Male	0.88	0.15	1.96	0.085
	Female	0.84	0.23		
Educational Status	Non-formal/Primary	0.83	0.22	-6.77	<0.001
	Others*	0.94	0.09		
Occupational Status	Working	0.91	0.12	4.49	<0.001
	Not working	0.83	0.22		
Marital Status	Married	0.87	0.17	1.78	0.005
	Single/Widow/Widower/Divorced	0.78	0.33		
Socio-Economic Status (Modified BG Prasad Scale)	Class I, II, III	0.91	0.1	4.6	<0.001
	Class IV, V	0.83	0.23		
Living Status	With family/Non-family members	0.86	0.20	0.32	0.684
	Alone	0.84	0.24		
Alcohol	Yes	0.84	0.16	-1.36	0.282
	No	0.87	0.21		
Smoking	Yes	0.86	0.15	0.00	0.842
	No	0.86	0.21		
Duration of Non-communicable Disease Morbidity	≤ 3 years	0.92	0.10	6.25	<0.001
	> 3 years	0.80	0.24		

Table 4 represents the multiple regression analysis. To identify the most significant predictors of the EQ Index score, all variables that were statistically significant in the univariate analysis (p < 0.05) were included in a multiple linear regression model. The final model identified several significant independent predictors of a lower EQ Index score. These included age ≥ 60 years (β = -0.290, p < 0.001), being single/widowed/divorced (β = -0.095, p = 0.047), having non-formal/primary education (β = -0.110, p = 0.035), and a disease duration of > 3 years (β = -0.221, p < 0.001). The R² of this model was 0.231, indicating that the independent variables explained 23.1% of the variability in the dependent variable. The overall regression model was statistically significant (F(6, 357) = 17.78, p < 0.001), explaining 23.1% of the variability in the EQ Index score (R² = 0.231; Adjusted R² = 0.218).

**Table 4:** Multiple Linear Regression to Predict the Factors Influencing EQ Index Value

Variable	Unstandardized Coefficients Beta	Standardized Coefficients Beta	Significance	95% Confidence Interval of B	
Constant	1.006	1.006	0.000	0.964	1.047
Age (≥60 years)	-0.126	-0.290	0.000	-0.169	-0.084
Marital Status (Single, Widow, Widower, Divorced)	-0.058	-0.095	0.047	-0.115	-0.001
Educational Status (Non-formal/Primary)	-0.048	-0.110	0.035	-0.093	-0.003
Occupational Status (Not Working)	-0.021	-0.051	0.312	-0.062	0.020
Socio-Economic Status (Lower, Lower Middle)	-0.010	-0.024	0.650	-0.052	0.033
Duration of Morbidity (>3 years)	-0.088	-0.221	0.000	-0.127	-0.050

## Discussion

Around 364 participants were recruited in the study, and HRQoL was assessed using the EQ-5D-5L study tool. The mean EQ index score was 0.86 ± 0.19, indicating a better quality of life. In our study, on the analysis of various domains of HRQoL, it showed that the majority of the participants were suffering from pain/discomfort (228, 62.6%). Similarly, the study conducted by Jyani et al. [13] among the general population in India showed that around 55% were suffering from pain/discomfort, whereas in another study conducted by Rajendran et al. [14], close to our geographical location (Chennai), among patients with end-stage renal disease, 65% had pain/discomfort, and among breast cancer patients in Mumbai [15], around 74.4% had pain/discomfort. This evidence suggests that, in comparison to the general population, the pain/discomfort domain is affected more among NCD patients. This high prevalence of pain/discomfort may stem from a combination of factors, including the direct pathological effects of chronic diseases, limited access to specialized pain management clinics in semi-urban settings, and a potential over-reliance on intermittent, non-prescribed analgesics rather than structured pain control regimens.

Self-care was also a key factor in determining the HRQoL among NCD patients. In this study, more than one-third (38.4%) of the participants had a problem in self-care. A study by Kaur et al. [16] in North India among diabetes patients also indicates that around 38.6% of the participants had problems in self-care. Empowering self-care in NCD patients will have its impact on quality of life through better control of their conditions, following best practices, adhering to preventive measures, early identification of complications, and appropriate follow-up that enhances their well-being [17].

The overall quality of life was represented by the EQ utility index, and it was 0.86 ± 0.19. When compared internationally, this HRQoL score is slightly higher than that reported among NCD patients in some other low- and middle-income countries, such as a study in Ethiopia, which found a mean EQ index of 0.78 among diabetic patients, but comparable to findings in some Asian settings, like Indonesia [18,19]. This suggests that, while the burden on quality of life is substantial, local healthcare and social support structures may play a role in mitigating its severity. In the study by Sholihat and Utami [18], among patients undergoing chronic disease management (diabetics and hypertensives), it was found to be 0.88 ± 0.11. The consistency of this finding with the Sholihat and Utami study validates our result and points to a similar, quantifiable impact of managing chronic diseases like diabetes and hypertension on patients’ quality of life, and also suggests that the EQ-5D-5L instrument consistently captures a common level of functional impairment experienced by patients managing these prevalent NCDs.

Our regression analysis confirmed that age ≥60 years was a significant predictor of poorer HRQoL (p < 0.05). These associations may stem from physical degeneration and the psychosocial void linked to a lack of familial or social support. This is in line with the study by Aschalew et al. [19] among diabetes mellitus patients, which found that the older age group and being single had a significant association with HRQoL. This association may stem from the aging process itself, where progressive degenerative changes can worsen the severity and functional impact of chronic conditions, leading to a decline in HRQoL [20]. Similarly, marital status was a key factor; participants who were single, widowed, or divorced reported significantly lower HRQoL scores, which is consistent with literature suggesting that the absence of a partner can correlate with reduced emotional and social support [21], which is a critical buffer against the psychological burden of chronic illness.

In the study by Soji et al. [22] in Kerala among diabetics, it was indicated that those with less education had higher odds of poor HRQoL, which is similar to our finding that those with non-formal or primary school education had lower HRQoL when compared to participants with higher levels of education. Health literacy influences self-care capacity, treatment adherence, and timely help-seeking behavior. Therefore, integrating community-based education interventions is critical. Disease duration >3 years significantly affected EQ scores, likely due to cumulative physical decline and emotional fatigue. This underlines the importance of long-term patient engagement and regular monitoring within NCD care frameworks.

The main strengths of the study were using a standardized study tool in assessing HRQoL and conducting it in a community setting. Whereas the limitation of the study was the lack of representation of all types of NCDs. The sampling frame is limited to program-enrolled households, potentially introducing selection bias. Future studies should consider multi-stage cluster sampling to improve representativeness across socio-economic strata. No adjustments were made for potential confounders, such as comorbidities, treatment adherence, or healthcare access. This omission may limit interpretability. Though EQ-5D-5L is widely validated, its application in Indian contexts may be influenced by cultural stoicism and underreporting of mental health issues, which can skew responses - particularly in domains like anxiety/depression and self-care. Tailoring interpretations to cultural contexts is essential. Our analysis focused on socio-demographic and disease-related factors and did not include other important lifestyle variables known to influence HRQoL, such as physical activity levels, dietary habits, or social support networks. Future studies should aim to incorporate these factors for a more comprehensive model.

## Conclusions

This study revealed that a significant proportion of NCD patients in this rural district in Tamil Nadu experienced compromised quality of life, particularly in domains like pain and self-care. Poor HRQoL was strongly associated with older age, low education, single marital status, and prolonged disease duration. These findings reinforce the need to incorporate HRQoL monitoring as a routine part of NCD care under national programs. Future NCD programs in Tamil Nadu and similar settings should incorporate culturally adapted pain management protocols, psychosocial support for elderly and isolated individuals, targeted health literacy initiatives, and community-based chronic disease counseling. Specific interventions could include mobile health apps to reinforce health literacy, peer-support groups for the elderly and widowed to mitigate social isolation, and integrating regular pain assessment and management protocols into primary health care visits for NCD patients. These measures could substantially enhance the well-being of NCD patients in resource-constrained environments.

## Appendices

Questionnaire

* Indicates required question

CONSENT:

1. Purpose of the Study: The purpose of this study is to analyse the quality of life among patients with any Non-Communicable Disease.

Participation: Your participation in this study is entirely voluntary. You may choose not to participate, and you may withdraw from the study at any time without any negative consequences.

Confidentiality: All information you provide will be kept confidential. Your responses will be anonymized and will not be linked to your identity or you e-mail id. The data collected will be used solely for research purposes.

Risks and Benefits: There are no known risks associated with participation in this study. The results of this study may lead to improvements in enhancing the quality of healthcare services.

By clicking "I Agree" below, you acknowledge that you have read and understood the information provided and that you consent to participate in this study.

Mark only one circle.

○ I Agree

Socio Demographic Profile:

2. NAME *

3. AGE *

4. SEX *

*Mark only one circle.* ○ Male ○ Female ○ Others

5. PLACE OF RESIDENCE:*

6. MARITAL STATUS *

*Mark only one circle*. ○ Single ○ Married ○ Divorced ○ Widow ○ Widower ○ Separated

7. EDUCATIONAL STATUS *

*Mark only one ciricle*. ○ Non Formal Education ○ Primary Education ○ Secondary Education ○ Higher Secondary Education ○ Undergraduate Degree ○ Postgraduate Degree or Higher

8. OCCUPATION *

*Mark only one ciricle*. ○ Not Working ○ Home Maker ○ Student ○ Employed ○ Self-Employed ○ Retired

9. MONTHLY HOUSEHOLD INCOME (INR)* ___________________

10. TYPE OF RESIDENCE *

*Mark only one circle*. ○ Urban ○ Rural

11. LIVING ARRANGEMENT *

*Mark only one circle.* ○ With Family ○ Living Alone ○ With Non-Family members

12. TOTAL NUMBER OF FAMILY MEMBERS:* _______________

13. UNIQUE ID:* _____________

Non-Communicable Disease:

14. SMOKING STATUS*

*Mark only one circle.* ○ Never smoked ○ Previous smoker ○ Current smoker

15. DURATION OF SMOKING: (If absent, enter NO)*

16. ALCOHOL CONSUMPTION*

*Mark only one circle*. ○ Never drinms ○ Occasionally drinks ○ Frequently drinks

17. DURATION OF ALCOHOL CONSUMPTION: (If absent enter NO)*

18. PRESENCE OF COMORBIDITIES

Mark only one circle. ○ Diabetes ○ Hypertension ○ Cardiovascular disease ○ Respiratory disease

19. DURATION OF COMORBIDITY: * _______________

EQ 5D 5L Descriptive System:

20. MOBILITY *

*Mark only one circle.* ○ I have no problems walking about. ○ I have slight problems walking about. ○ I have moderate problems walking about ○ I have severe problems walking about ○ I am unable to walk about

21. SELF-CARE: *

*Mark only one circle.* ○ I have no problems washing or dressing myself. ○ I have slight problems washing or dressing myself. ○ I have moderate problems washing or dressing myself. ○ I have severe problems washing or dressing myself. ○ I am unable to wash or dress myself.

22. USUAL ACTIVITIES (work, study, house work, leisure activities): *

*Mark only one circle.* ○ I have no problems doing my usual activities ○ I have slight problems washing or dressing myself. ○ I have moderate problems washing or dressing myself. ○ I have severe problems washing or dressing myself. ○ I am unable to do my usual activities

23. PAIN/DISCOMFORT: *

*Mark only one circle*. ○ I have no pain or discomfort ○ I have slight pain or discomfort ○ I have moderate pain or discomfort ○ I have severe pain or discomfort ○ I have extreme pain or discomfort

24. ANXIETY/DEPRESSION: *

*Mark only one circle*. ○ I am not anxious or depressed. ○ I am slightly anxious or depressed. ○ I am moderately anxious or depressed. ○ I am severely anxious or depressed. ○ I am extremely anxious or depressed.

25. EQ VISUAL ANALOGUE SCALE: (0 TO 100) - How healthy are you feeling today? *
